# Major Soybean Maturity Gene Haplotypes Revealed by SNPViz Analysis of 72 Sequenced Soybean Genomes

**DOI:** 10.1371/journal.pone.0094150

**Published:** 2014-04-11

**Authors:** Tiffany Langewisch, Hongxin Zhang, Ryan Vincent, Trupti Joshi, Dong Xu, Kristin Bilyeu

**Affiliations:** 1 Plant Genetics Research Unit, United States Department of Agriculture-Agricultural Research Service, University of Missouri, Columbia, Missouri, United States of America; 2 Department of Computer Science, University of Missouri, Columbia, Missouri, United States of America; 3 Division of Computing, McKendree University, Lebanon, Illinois, United States of America; 4 National Center for Soybean Biotechnology, University of Missouri, Columbia, Missouri, United States of America; 5 Informatics Institute, University of Missouri, Columbia, Missouri, United States of America; 6 Christopher S. Bond Life Sciences Center, University of Missouri, Columbia, Missouri, United States of America; Nanjing Agricultural University, China

## Abstract

In this Genomics Era, vast amounts of next-generation sequencing data have become publicly available for multiple genomes across hundreds of species. Analyses of these large-scale datasets can become cumbersome, especially when comparing nucleotide polymorphisms across many samples within a dataset and among different datasets or organisms. To facilitate the exploration of allelic variation and diversity, we have developed and deployed an in-house computer software to categorize and visualize these haplotypes. The SNPViz software enables users to analyze region-specific haplotypes from single nucleotide polymorphism (SNP) datasets for different sequenced genomes. The examination of allelic variation and diversity of important soybean [*Glycine max* (L.) Merr.] flowering time and maturity genes may provide additional insight into flowering time regulation and enhance researchers' ability to target soybean breeding for particular environments. For this study, we utilized two available soybean genomic datasets for a total of 72 soybean genotypes encompassing cultivars, landraces, and the wild species *Glycine soja*. The major soybean maturity genes *E1*, *E2*, *E3*, and *E4* along with the *Dt1* gene for plant growth architecture were analyzed in an effort to determine the number of major haplotypes for each gene, to evaluate the consistency of the haplotypes with characterized variant alleles, and to identify evidence of artificial selection. The results indicated classification of a small number of predominant haplogroups for each gene and important insights into possible allelic diversity for each gene within the context of known causative mutations. The software has both a stand-alone and web-based version and can be used to analyze other genes, examine additional soybean datasets, and view similar genome sequence and SNP datasets from other species.

## Introduction

With the advent of next-generation sequencing and large-scale online databases, biologists are becoming increasingly inundated with the vast amounts of genomic data available and limited bioinformatics support for determining the best way to analyze and extract useful information. Unlike previous times, scientists now have a wealth of publically-available databases at their fingertips. The accelerating release of next-generation sequencing data and annotated genomes provides the opportunity to test new hypothesis before even turning to the lab bench. Taking advantage of the abundance of SNPs found in related genomes can be a powerful resource for investigating allelic variation and diversity in genomic regions and particular genes of interest.

To aid in this endeavor, we have developed SNPViz, a simple-to-use SNP haplotype viewer. SNPViz provides an array of options to view haplotypes from specific gene regions as well as much larger chromosomal regions. Users are able to input one or more SNP-array files along with the option of including an annotation file. The software clusters the samples and creates a phylogeny based on the identity of the haplotypes. The SNPs from the chosen region are displayed as a clustering pictorial, where haplotypes can be viewed as a basic classification of three colors representing SNPs identical to the reference, SNPs different from the reference, and positions with no SNP data. Alternatively, users can view this same SNP information with each nucleotide being signified by a different color. Plus, the nucleotide base can be included on the image. This highly visual program will facilitate research in mining for alleles and understanding allelic diversity.

We are interested in applying SNPViz to explore variation and diversity of alleles of genes controlling important traits in soybean. Soybean [*Glycine max* (L.) Merr.] is classified as a short day plant, and it contains the classical maturity loci *E1* [1], *E2* [1], *E3* [2], *E4* [3] as well the plant architecture gene *Dt1* [4]. We can now take advantage of these recently cloned genes, *E1* [5], *E2* [6], *E3* [7], *E4* [8] and *Dt1* [9],[10], whose genomic position and sequence are known (Table 1).

**Table 1:** Major flowering time/maturity genes present in the Williams 82 reference sequence and positions used around those genes for haplotype analysis.

Gene	Reference Name	Function or Orthologue	Gene Location (from GFF)	Haplotype Region
*e1-as* 1	Glyma06g23026	B3 DNA binding protein	20006928–20007814 bp	20003428–20011314 bp
*E2* 2	Glyma10g36600	(*At*) GIGANTEA	44716804–44738165 bp	44713304–44741665 bp
*E3* 2	Glyma19g41210	Phytochrome A	47512505–47519937 bp	47509005–47523437 bp
*E4* 2	Glyma20g22160	Phytochrome A	32087661–32093266 bp	32084161–32096766 bp
*Dt1* 2	Glyma19g37890	(*At*) TERMINAL FLOWER1	44979743–44981385 bp	44976243–44984885 bp

1 Gene location based on the Williams 82 reference sequence Glyma1.1.

2 Gene location based on the Williams 82 reference sequence Glyma1.0.

Alleles of the major maturity *E1* gene on chromosome six were recently cloned and revealed to encode a transcription factor that is distantly related to the B3 superfamily (Table 1) [5]. The *E1* locus (Glyma06g23026) has four known alleles with one fully functional allele, *E1* [5] (Table 2). The *e1-as* allele, present in the reference sequence of the cultivar ‘Williams 82,’ has a defining missense mutation where a glycine (G) to cytosine (C) nucleotide substitution at the 44 base pair (bp) position of the coding sequence results in the amino acid residue at position 15 being changed from an arginine to a threonine (R15T) [5],[11],[12]. The *e1-as* allele conditions earlier flowering time and maturity compared to the *E1* allele [5]. The *e1-null* allele is a deletion of the entire gene while *e1-fs* is defined by a single-base deletion in the coding sequence at position 48 bp. This deletion causes a frameshift and ultimately a truncated peptide [5]. Other polymorphisms around the *E1* gene were defined, but these changes were not proposed to be involved in gene function [5]. Recently, the *e1-re* and *e1-p* alleles were described that have polymorphic changes outside the gene region but have no effect on the protein. The *e1-re* allele has a long interspersed nuclear element (LINE) that is inserted in the *E1* promoter region while *e1-p* has an altered 5′ upstream region [13]. While *e1-re* and *e1-p* represent additional polymorphic alleles of *E1*, they have not yet been fully genetically characterized for their effects on flowering time.

**Table 2:** Reported polymorphic alleles of major maturity genes with reference to the Williams 82 sequence.

Allele	Polymorphism compared to Williams 82	Protein Change	Reference
*E1* 1	SNP: C/G (Gm06:20007173)	R15T	[5]
*e1-fs* 2	Single base deletion: A/- (Gm06:20007177)	Frameshift after AA 15	[5]
*e1-nl*	Entire gene deleted	No protein	[5]
*e2*	SNP: A/T (Gm10:44732850)	K521*	[6]
*E3 (short)* 3	Deletion of 2633 bp in intron 3 (Gm19: 47516697-47519330)	No change	[7]
*e3*	Deletion of 15502 bp (Gm19: 47531605-47516603)	Frameshift after AA 1060	[7]
*e3-ft3*	SNP: G/A (Gm19: 47516339)	G1050R	[7]
*e4 (SORE-1)*	Insertion of 6238 bp retrotransposon (Gm20: 32089086)	Frameshift after AA 231	[8]
*e4-oto*	Single-base deletion: G/- (Gm20: 32089731)	Frameshift after AA 446	[15]
*e4-tsu*	Single-base deletion: T/- (Gm20: 32091045)	Frameshift after AA 749	[15]
*e4-kam*	Single-base deletion: G/- (Gm20: 32091473)	Frameshift after AA 892	[15]
*e4-kes*	Single-base deletion: A/- (Gm20: 32091662)	Frameshift after AA 954	[15]
*dt1* (R62S)	SNP: C/A (Gm19: 44981190)	R62S	[9]
*dt1* (P113L)	SNP: G/A (Gm19: 44980245)	P113L	[9]
*dt1* (R130K)	SNP: C/T (Gm19: 44980194)	R130K	[9]
*dt1* (R166W)	SNP: T/A (Gm19: 44980087)	R166W	[9]

1 Uppercase allele designations indicate the dominant functional versions of the gene. In each case, the recessive mutant version of the gene is earlier flowering and maturing than the functional dominant version of the gene. The Williams 82 genome contains an earlier maturing missense version of *E1* (*e1-as*; T15R compared to the wild-type functional *E1*) [5]. Allele names are taken or modified from the published descriptions for clarity.

2 The underlined alleles were identified and described in the literature but were not present in the two datasets used for this analysis.

3 Although the Williams 82 *E3* allele is considered functional, it was shown to contain an insertion in intron three consisting of transposable element-like sequences when compared to other functional *E3* alleles without the insertion in intron 3 [7]. We herein denote the *E3* from Williams 82 as *E3* and the equivalently functional shorter *E3* allele as *E3 (short)*.

*E2* (*GmGIa*, Glyma10g36600) has high homology to the Arabidopsis GIGANTEA protein, which is involved in the circadian clock mechanism of the flowering time pathway [6]. This gene has been mapped to the long arm of chromosome ten, spans a 21.5-kilobase (kb) region, and has 14 exons [6]. Originally, three nonsynonymous SNPs were reported within the exons, but only a SNP in exon ten resulted in a functional change (Table 2). Within exon ten, a nonsense mutation occurs when thymine (T) is substituted for adenine (A) at base 1561 of the coding sequence in the *e2* allele [6]. This premature stop codon truncates the 1170 amino acid protein to 521 amino acids [6]. This nonfunctional *e2* allele has an early flowering phenotype compared to *E2* [6]. Additional polymorphisms in and around the *E2* gene were subsequently described, but these alleles were classified as functional [13].

*E3* (GmPhyA3, Glyma19g41210), a phytochrome A gene, is a photoreceptor in the flowering time pathway that delays flowering under long-day conditions with high red/far-red light intensity [7],[14]. This gene has been mapped to chromosome 19 and is responsible for a major maturity effect [7]. *E3* has two functional alleles, designated here as *E3* and *E3 (short)* (Table 2). Both of these alleles have all four exons and encode identical protein sequences, but *E3*, which is present in the Williams 82 genome, has an insertion within the last intron compared to *E3 (short)*. No evidence exists to differentiate functionally between the *E3* and *E3 (short)* alleles [7]. The null *e3* allele has a 13-kb region deleted from the third intron that excludes the entire fourth exon compared to *E3 (short)* [7]. A second mutant *e3-ft3* allele was described that contains a missense mutation substituting a neutral amino acid for a charged one in a conserved amino acid (G1050R) [7] (Table 2).

Another phytochrome A gene, *E4* (GmPhyA2, Glyma20g22160), has been cloned, mapped to chromosome 20, and has several variant alleles [8],[15]. This gene has one functional *E4* allele and five dysfunctional alleles (Table 1). The nonfunctional *e4* (*SORE-1*) allele is caused by the insertion of a 6,238 bp *Ty1/copia*-like retrotransposon in exon one [8]. This insertion introduces a premature stop codon that truncates the 1123 amino acid protein to 237 amino acids [8] (Table 2). The other four dysfunctional alleles, *e4-oto*, *e4-tsu*, *e4-kam*, and *e4-kes*, each have one deleted nucleotide in the gene-coding region, which creates a frameshift and truncated protein of 456 amino acids, 759 amino acids, 894 amino acids, and 979 amino acids, respectively [15] (Table 2).

The indeterminate gene *Dt1* controls stem growth habit [16]. The indeterminate allele *Dt1* has four exons and encodes a homologue of the Arabidopsis regulatory protein TERMINAL FLOWER1 (TFL1) [9],[10]. Functional *Dt1* alleles promote continued stem growth after flowering while determinate mutant *dt1* alleles condition abrupt stem termination after flowering. Four missense *dt1* alleles—*dt1* (R62S), *dt1* (P113L), *dt1* (R130K), and *dt1* (R166W)—have been identified that are responsible for the determinate plant architecture phenotype [9],[10] (Table 2).

The aim of this study was to utilize the new SNPViz software to determine the number of major haplotypes for each of these genes in two soybean datasets containing cultivated and wild-type soybean accessions. We also examined the consistency of the haplotypes with characterized variant alleles and looked for evidence of artificial selection. The SNPViz software is not exclusive to soybean and can be used to analyze different genes from multiple species.

## Materials And Methods

### Development Of Snpviz

SNPViz is created to be an easy-to-use haplotype visualization tool that quickly clusters regions of multi-sampled SNP dataset(s) into haplotypes. This program has the ability to display different pictorial representations of SNP data along with the gene and exon locations found within that region. SNPViz treats the SNP-array data as a special kind of multiple sequence alignment subject to Clustal analysis. Since base positions with identical sequence are not included in the data files, the phylogenetic tree is generated by calculating sequence identity. Visualization for each genome is indicated by sequence code or nucleotide base compared to the reference sequence at each variable nucleotide position on the chromosome.

SNPViz is available as a Java stand-alone package and a web-based tool (http://soykb.org/SNPViz) incorporated into Soybean Knowledge Base (SoyKB, http://soykb.org) [17]. Both versions of this program have a free non-commercial research-use license and can be run on Windows, Mac OS, and Linux. This web-based program can be run on any Internet browser except for Google Chrome for Mac because it does not support Java. Both programs can be run with Java 7, the most recent version, but Mac users should also update the Java Development Kit and Java Runtime Environment. For the web-based version, users can contact SoyKB to deposit their datasets into SoyKB so that they can view their data along with the publicly-available SNP-array and genotype-by-sequencing (GBS) soybean datasets at SoyKB. Users can more easily input their datasets directly with the stand-alone version. When the stand-alone version is downloaded from SoyKB, SNP files for the publicly-available resequencing of 31 wild and cultivated soybeans [18] and whole-genome sequencing of *Glycine soja* Sieb. and Zucc. [19] are included.

### Input Options

To run SNPViz, a SNP-array or GBS file must be inputted as a tab-delimited text file. The first line of this file should include SNP data and not column identifiers. The chromosome number must be in the first column and the sequence position in the second column. The software assumes that the first sample column is the reference sequence. The remaining columns should contain all sample SNP data as either a single nucleotide or a double nucleotide. The double nucleotide will be converted into the International Union of Pure and Applied Chemistry (IUPAC) single letter code [20]. SNPViz can be utilized to study SNP data from individual chromosomes, and data can be uploaded for one chromosome at a time.

SNPViz has several useful features including functions to input datasets, modify sample names, and delete unwanted datasets. New datasets can be added to the program by uploading a header file, which contains all the sample names. This header file is stored as a XML file, and SNPViz links this header file to your inputted SNP-array or GBS files. The header file and SNP text files must be in the same folder, and this folder must have the same name as the dataset, Once the header file has been added, the sample names, as indicated in the header file, will automatically appear in the SNPViz clustering window when a SNP text chromosome file is inputted. Users will then be able to select which samples to include in the pictorial clustering image. Users also have the option to modify the sample names or delete datasets that have already been inputted into the program.

A general feature format (GFF3/GFF2) or general transfer format (GTF) annotation file can be included so that SNP positions can be correlated with gene regions and exon locations. Once the GFF/GTF file is inputted, a pop-up window will appear and prompt the user to select an identification feature of the GFF file, such as name, locus_id, or mRNA, so the program can identify which label corresponds with the gene and exon locations. If a GFF/GTF file is unavailable or not provided by the user, gene positions will not be included in the output pictorial. For the web-based version incorporated in SoyKB, gene annotations for either Glyma1.0 or Glyma1.1 are automatically retrieved from the database.

When entering chromosome start and end positions, only an integer value will be accepted. Other formats will be rejected, and a warning message will appear. The size of the viewable and printable chromosome region will depend on the inclusion of an annotation file and available computer memory.

### Display Options

After inputting the SNP-array or GBS file from the appropriate chromosome, the annotation file (optional), and the nucleotide position start and end information, users can choose several options to display the output. The SNP data can be shown as three colors or multiple colors to represent each variant base position for each genome. With three colors, black indicates that a base at that position is different from the reference sequence while white signifies that the base at that position is identical to the reference. The nucleotide of the reference sequence is displayed by default. When the base at that position contains a “-” or “N” instead of a nucleotide, it is colored gray to represent no data/missing data. Alternatively, users can opt to visualize the SNP data with multi-colors. Red, blue, green, and yellow are used to represent A, G, C, and T, respectively. Other colors indicate when more than one nucleotide could occur at that position. Both the three- or multiple-colored option can be viewed with or without the nucleotide (Figure S1). An auto-defined width can be included between two neighboring samples for clarity if the user selects the “show space” option. These display options allow the user to examine their datasets in multiple visual ways.

### Phylogeny Clustering

To cluster samples into haplotype groups, the distance matrix data are initially calculated using ClustalW, as described in Thompson et al. 1994 [21]. The samples are grouped, and a phylogeny tree is constructed using Unweighted Pair Group Method with Arithmetic Mean (UPGMA) [22]. The Java code for these algorithms can be found at http://www.itu/dk/people/sestoft/bsa/Match7/java [22].

### Integration Of Datasets

The user can integrate up to three different datasets as long as the same chromosome SNP file is selected for each dataset. When multiple datasets are merged into one image, SNPViz will automatically assume that the first sample is the reference for each dataset. Also, for SNP positions from multiple datasets not present in one dataset but present in another dataset, the reference SNP is created for that position. During phylogeny tree construction, the genetic distance is described as the number of mismatched data points divided by the number of total data points [21]. Therefore, the SNP locations with no data, usually represented as “-” or “N”, are ignored when the phylogeny trees are constructed.

### Output Options

The clustering tree can be saved as a Joint Photographic Experts Group (JPEG/JPG) or portable network graphics (PNG) file. All the SNP positions within the clustering tree can be saved as a text file. This text file will also include the gene and exon information for the haplotype region if a GFF/GTF was used. The distance matrix information can also be saved as a text file.

### Snp Datasets

We examined SNP haplotypes in two soybean SNP datasets. The publicly-available resequencing of 31 wild and cultivated soybeans provided an excellent resource for examining allelic diversity between 17 wild (*Glycine soja*) lines and 14 cultivated (*Glycine max*) lines from various locations in China with one line each from Taiwan, Brazil, and the USA [18]. For the purpose of this publication, we refer to the 31 genomes data as the Chinese collection or lines. These lines were resequenced as 45-bp or 76-bp paired-end reads. All 901.75 million paired-end reads were mapped to the Williams 82 reference genome (Glyma1.01) with a 5× sequencing depth [12],[18]. Approximately 6.3 million SNPs were identified [18].

Sequencing of the Nested Association Mapping (NAM) parents offered a collection of 41 diverse soybean lines from North America. The NAM population consists of 17 high yielding parents from eight states, 15 lines with diverse ancestry, and eight plant introductions (B. Diers, personal communication 2013). The last sequenced line is the hub parent, the Iowa State University cultivar IA3023. These lines were sequenced as 150 bp paired-end reads and aligned to the Williams 82 whole genome sequence (Glyma1.01) [12] (P.Cregan and Q. Song, personal communication, 2013). Sequence coverage ranged from 4× to 15× among the parental lines. A total of 5.2 million variants, including ambiguous and missing calls, were identified.

### Definition Of Haplogroup

When analyzing the clustering pictorial output for a specific genic region, we identified haplotypes based on the first phylogeny tree branching point. In this first tier branching, haplotypes were clustered based on their sequence similarity or dissimilarity to the Williams 82 reference. We referred to these groupings as the Williams 82-like or non-Williams 82 haplogroup based on whether individual haplotypes were grouped with the reference or not. Identification of additional subgroups within these main haplogroups was possible if second or third tier branching points were considered.

### *E3* Genotyping Assay

A polymerase chain reaction (PCR)-based assay was developed to distinguish *E3*, *E3 (short)*, and *e3*. PCR primers were designed from Williams 82 genomic sequence and *E3 (short)* as well as *e3* sequences described in Watanabe et al. 2009 [7]. A forward primer (targeting the sequence within intron 3 upstream from either deletion event) E3f (5′-TGGAGGGTATTGGATGATGC-3′) and three reverse primers E3r (5′-GGAAAGAGAGACATGTAGTGAATGAA-3′), E3 short (5′-ATTCGAGGCAGAGGAAGACC-3′), and e3r (5′-TGCGGCAAGTTCAACAGATA-3′) were near the forward primer specifically for the *E3*, *E3 (short)*, and *e3* alleles, respectively. Reactions were carried out in 20 µL containing template, 0.5 µM each primer, buffer (40 mM Tricine-KOH [pH 8.0], 16 mM MgCl_2_, 3.75 µg mL^−1^ BSA), 5% DMSO, 200 µM dNTPs, and 0.2× Titanium Taq polymerase (BD Biosciences, Palo Alto, CA). The 20 µL PCR reactions were initially denatured at 95°C for 5 minutes and then were denatured at 95°C for 20 seconds, annealed at 60°C for 20 seconds, and elongated at 72°C for 20 seconds for 35 cycles. PCR products were then resolved on a 1.5% agarose gel. PCR product sizes of 322 bp, 492 bp, and 173 bp indicated *E3*, *E3 (short)*, and *e*3, respectively.

## Results

### *E1* Haplotype Characterization

We first investigated the allelic variation of *E1*, the major contributor to photoperiod sensitivity and plant maturity. In Glyma1.1, Glyma06g23026 is an 887 bp gene that encodes a 234 amino acid protein (http://www.phytozome.net/). In the early genome version Glyma1.0, *E1* was designated as Glyma06g23040, but the indicated gene and protein size are inconsistent with the published *E1* gene information [5]. Therefore, we examined SNP polymorphisms for *E1* and 3.5 kb surrounding the gene using the gene coordinates from Glyma1.1 (Table 1). This area included the *e1-as* causative SNP at 20,007,173 bp (Table 2). Only the *E1* and *e1-as* alleles were present in the Chinese collection of wild and cultivated lines and the NAM parents. No obvious evidence supported the existence of either the *e1-fs* or *e1-nl* alleles in the datasets, although SNP-array data is not the best option to identify single-base deletions or large-scale genomic deletions.

**Figure 1:**
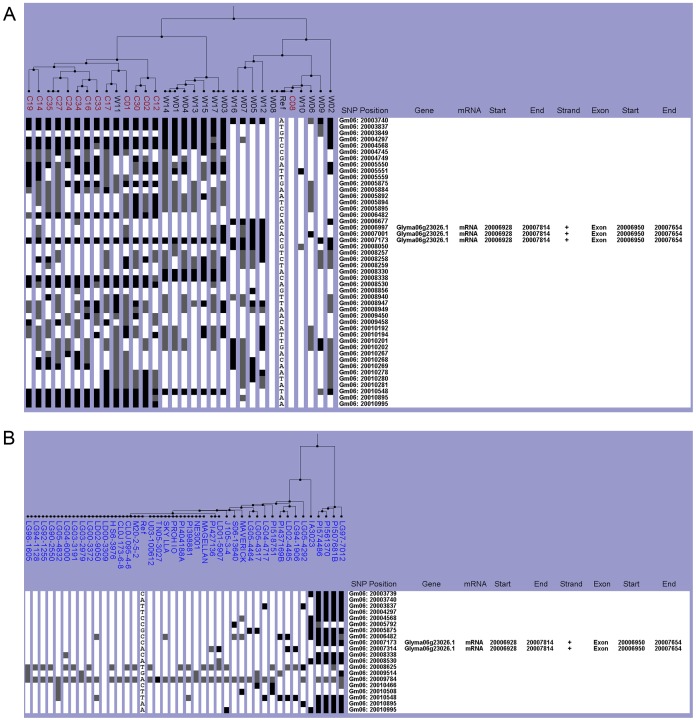
Haplotype analysis of the *E1* gene region. The SNPViz clustering pictorial displayed the SNPs in a 7.8-kb region on chromosome six, which included Glyma06g23026. UPGMA grouped samples by calculating their sequence identity to the reference, Williams 82. Each soybean line was represented by a column with nucleotides only shown for the reference. All base positions that are identical to the reference are white, those that are different are black, and positions with no data or missing data are gray. Since an annotation file was included in this analysis, the gene locations, DNA strand, and exon start and end site are shown to the right of SNP position. Nucleotide polymorphisms were examined in A) the wild (black) and cultivated (red) lines from the Chinese collection and B) the NAM parents (blue).

In the Chinese collection, the *E1* region was subdivided into two haplogroups based on whether or not the regions were very similar to the reference Williams 82 region. The Williams 82-like haplogroup included ten lines, which were all wild except for the cultivated line C08 (Figure 1A). The second haplogroup, which was distinctly different from the reference, had 13 cultivated and eight wild lines. Additional subgroupings within both haplogroups were evident (Figure 1A). All lines in the Chinese collection were classified as *E1* or *e1-as* according to their nucleotide (G or C) at the causative SNP. The wild lines W06, W08, and W10 and a single cultivated line C08 had the *e1-as* allele (Figure 1A, Tables 3 and 4). Even though ten lines were categorized in the Williams 82-like haplogroup, only these four lines contained the defining *e1-as* mutation, but these four lines were nearly identical to Williams 82 for this region.

**Table 3:** Maturity and growth determinate genotypes for wild soybean.

Accession^1^	Local Name	Description	Location	*E1/e1-as*	*E2/e2*	*Dt1/dt1*
W13	Yimeng15	Wild	Inner Mongolia, PRC	*E1*	*E2*	*Dt1*
W14	Yimeng19	Wild	Inner Mongolia, PRC	*E1*	*E2*	*Dt1*
W03	Wuhai4	Wild	Inner Mongolia, PRC	*E1*	*E2*	*Dt1*
W08	Jixian11	Wild	Heilongjian, PRC	***e1-as***	*E2*	*Dt1*
W06	Jidong5	Wild	Heilongjian, PRC	***e1-as***	*E2*	*Dt1*
W10	Shuangcheng4	Wild	Heilongjian, PRC	***e1-as***	*E2*	*Dt1*
W16	Hailun19	Wild	Heilongjian, PRC	*E1*	*E2*	*Dt1*
W02	Donggou16	Wild	Liaoning, PRC	*E1*	*E2*	*Dt1*
W09	Kaiyuan21	Wild	Liaoning, PRC	*E1*	*E2*	***dt1*** ** (R166W)**
W17	Zhangwu20	Wild	Liaoning, PRC	*E1*	*E2*	*Dt1*
W07	Fengcheng20	Wild	Liaoning, PRC	*E1*	*E2*	*Dt1*
W01	Beijing4	Wild	Beijing area, PRC	*E1*	*E2*	*Dt1*
W11	Taiyuan67	Wild	Shanxi, PRC	*E1*	***e2***	***dt1*** ** (R62S)**
W05	Mengjin1	Wild	Henan, PRC	*E1*	***e2***	*Dt1*
W04	Yanjin3	Wild	Henan, PRC	*E1*	*E2*	*Dt1*
W15	Yanjin4	Wild	Henan, PRC	*E1*	*E2*	*Dt1*
W12	Anqing18	Wild	Anhui, PRC	*E1*	*E2*	*Dt1*

1 *E3* and *E4* genotypes are not shown because the causative SNP was not identified in the data.

**Table 4:** Maturity and growth determinate genotypes for cultivated soybean.

Accession^1^	Local Name	Description	Location	*E1/e1-as*	*E2/e2*	*Dt1/dt1*
C33	Hefeng25	Advanced bred line	Heilongjian, PRC	*E1*	***e2***	*Dt1*
C19	Jilinxiaoli1	Landrace	Jilin, PRC	*E1*	***e2***	*Dt1*
C02	Tiefeng8	Advanced bred line	Liaoning, PRC	*E1*	***e2***	***dt1*** ** (R166W)**
C27	Cangdou5	Advanced bred line	Hebei, PRC	*E1*	***e2***	*Dt1*
C12	Jindou21	Advanced bred line	Shanxi, PRC	*E1*	***e2***	*Dt1*
C01	Wenfeng7	Advanced bred line	Shandong, PRC	*E1*	***e2***	***dt1*** ** (R62S)**
C30	Yudou12	Advanced bred line	Henan, PRC	*E1*	***e2***	***dt1*** ** (R62S)**
C17	Zigongdongdou	Landrace	Sichuan, PRC	*E1*	*E2*	***dt1*** ** (P113L)**
C24	Gandou4	Advanced bred line	Jiangxi, PRC	*E1*	***e2***	***dt1*** ** (P113L)**
C34	Gui199	Landrace	Guangxi, PRC	*E1*	***e2***	*Dt1*
C35	Guangzhoudali	Landrace	Guangdong, PRC	*E1*	***e2***	***dt1*** ** (P113L)**
C14	Brazil10	Advanced bred line	Brazil	*E1*	*E2*	***dt1*** ** (R166W)**
C16	Tainong1	Japan neutron-mutated	Taiwan	*E1*	*E2*	*Dt1*
C08	Union	Advanced bred line	USA	***e1-as***	*E2*	*Dt1*

1 *E3* and *E4* genotypes are not shown because the causative SNP was not identified in the data.

For the NAM parents, both the Williams 82-like and non-Williams 82 haplogroups had almost no SNP variation among the samples within each respective haplotype. The first haplogroup included four lines, LG97-7012, PI507681B, PI561370, and PI574486, which all had the *E1* base at the causative SNP at position 20,007,173 (Figure 1B, Table 5). The remaining 37 lines classified in the Williams 82-like haplogroup were *e1-as* as defined by position 20,007,173 (Figure 1B, Table 5).

**Table 5:** Maturity and growth determinate genotypes for the 41 NAM parents.

Strain^1^	*E1/e1-as*	*E2/e2*	*E3/e3*	*Dt1/dt1*
IA3023	***e1-as***	*E2*	*E3*	*Dt1*
SKYLLA	***e1-as***	*E2*	***e3***	*Dt1*
U03-100612	***e1-as***	*E2*	***e3***	*Dt1*
LG98-1605	***e1-as***	***e2***	*E3 short*	*Dt1*
LG94-1128	***e1-as***	***e2***	*E3*	*Dt1*
PI437169B	***e1-as***	***e2***	*E3*	*Dt1*
PI518751	***e1-as***	***e2***	*E3*	*Dt1*
S06-13640^2^	***e1-as***	*E2*		*Dt1*
LG05-4317	***e1-as***	*E2*	*E3 short*	*Dt1*
4J105-3-4	***e1-as***	*E2*	*E3*	*Dt1*
LD00-3309	***e1-as***	*E2*	*E3*	*Dt1*
LD02-4485	***e1-as***	*E2*	*E3*	*Dt1*
MAGELLAN	***e1-as***	*E2*	*E3*	*Dt1*
MAVERICK	***e1-as***	*E2*	*E3*	*Dt1*
LG03-2979	***e1-as***	*E2*	*E3*	*Dt1*
LG03-3191	***e1-as***	*E2*	*E3*	*Dt1*
LG90-2550	***e1-as***	*E2*	*E3*	*Dt1*
LG92-1255	***e1-as***	*E2*	*E3*	*Dt1*
LG04-6000	***e1-as***	*E2*	*E3*	*Dt1*
IA3023	***e1-as***	*E2*	*E3*	*Dt1*
LD01-5907	***e1-as***	*E2*	*E3*	*Dt1*
LG04-4717	***e1-as***	*E2*	*E3*	*Dt1*
LG05-4464	***e1-as***	*E2*	*E3*	*Dt1*
LG94-1906	***e1-as***	*E2*	*E3*	*Dt1*
LG00-3372	***e1-as***	*E2*	*E3*	*Dt1*
TN05-3027	***e1-as***	*E2*	*E3*	*Dt1*
5M20-2-5-2	***e1-as***	*E2*	*E3*	*Dt1*
C10J095-4-6	***e1-as***	*E2*	*E3*	*Dt1*
NE3001	***e1-as***	*E2*	*E3*	*Dt1*
LG05-4292	***e1-as***	*E2*	*E3*	*Dt1*
PI398881	***e1-as***	*E2*	*E3*	*Dt1*
PI404188A	***e1-as***	*E2*	*E3*	*Dt1*
CL0J173-6-8	***e1-as***	*E2*	*E3*	*Dt1*
HS6-3976	***e1-as***	*E2*	*E3*	*Dt1*
PI427136	***e1-as***	*E2*	*E3*	*Dt1*
LD02-9050	***e1-as***	*E2*	*E3*	*Dt1*
PROHIO	***e1-as***	*E2*	*E3*	*Dt1*
LG05-4832	***e1-as***	*E2*	*E3*	*Dt1*
PI507681B	*E1*	***e2***	***e3***	*Dt1*
LG97-7012	*E1*	***e2***	*E3*	*Dt1*
PI561370	*E1*	***e2***	*E3*	*Dt1*
PI574486	*E1*	***e2***	*E3*	*Dt1*

1 The *E4* genotype is not shown because the causative SNP was not identified in the data.

2 DNA was unavailable for *E3/e3* genotyping.

Comparison of both datasets revealed *E1* allelic diversity between Chinese and American soybean lines. Within the Chinese collection only four lines had the *e1-as* allele, but the United States NAM lines were completely *e1-as* except for LG97-7012, PI507681B, PI561370, and PI574486 (Table 5). When these two datasets were integrated and viewed with SNPViz, all *e1-as* lines are categorized as one haplotype group (Figure S2). Interestingly, unlike the Chinese cultivated lines, the NAM parents purposely have been selected for similar maturity and high-yield capacity.

### *E2* Haplotype Characterization

Allelic variation of *E2*, an orthologue of GIGANTEA, was examined because *E2* may play a role in the flowering time pathway in a similar manner as GIGANTEA. Glyma10g36600 is a 21.5-kb gene with 14 exons and encodes an 1170 amino acid protein (Glyma1.1, http://www.phytozome.net/) [6]. Nucleotide polymorphisms were compared between the Chinese and NAM lines for this genic region along with 3.5 kb around the gene (Table 1). The causative difference between the *E2* and *e2* alleles is an A/T substitution at base position 44,732,850 (Table 2). Our collections included both of these alleles (Table 3). Haplogroups were determined by looking at the entire region (44,713,304–44,741,665 bp) while allele genotypes were assigned according to the nucleotide at the causative SNP.

**Figure 2:**
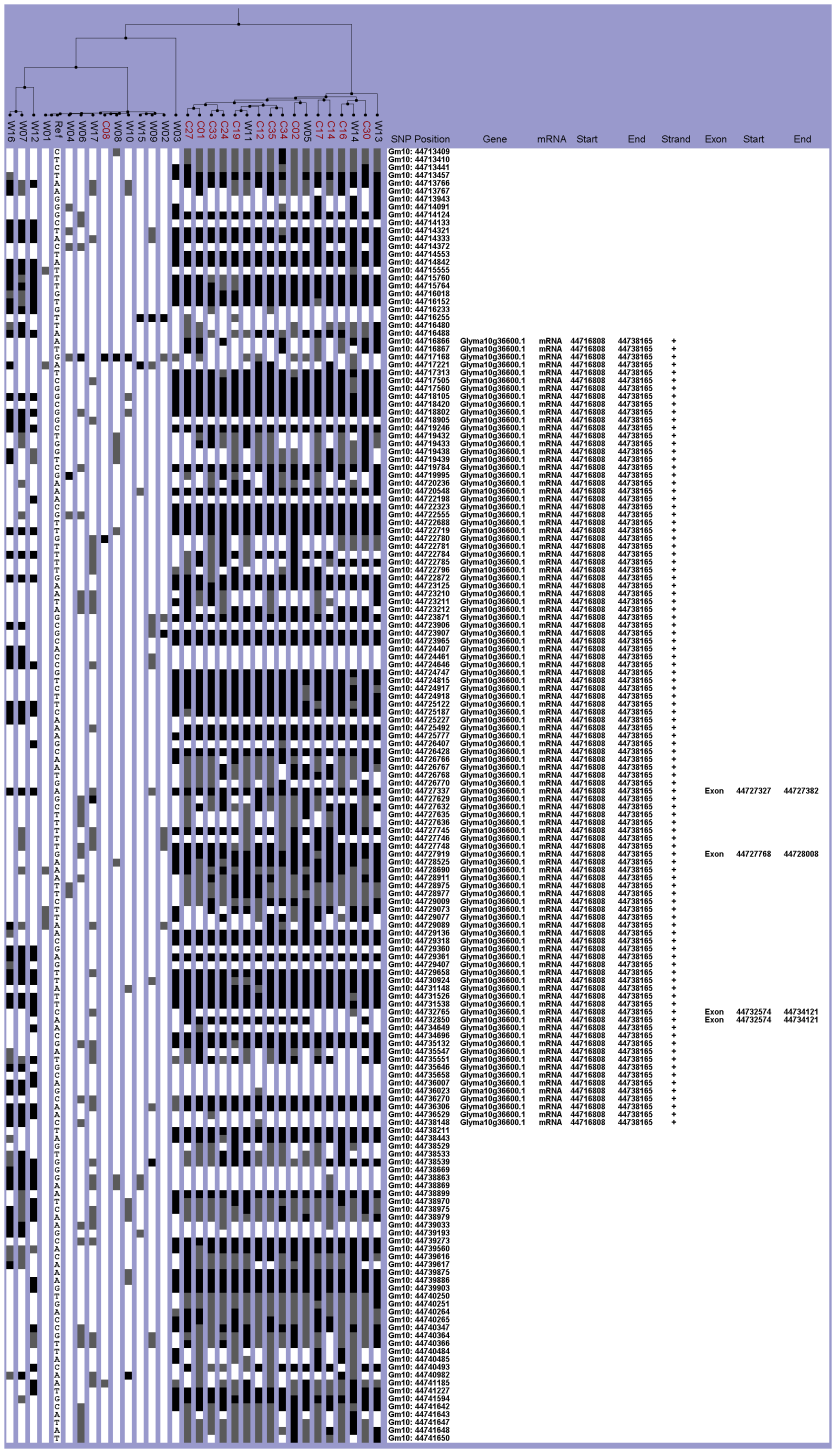
Haplotype analysis of the *E2* gene region for the Chinese collection. The SNPViz clustering pictorial displayed the SNPs in a 28.3-kb region on chromosome ten, which included Glyma10g36600. Nucleotide polymorphisms were examined in the wild (black) and cultivated (red) lines from the Chinese collection.

In the Chinese collection, two haplogroups were easily distinguished by their similarity and differences to the reference Williams 82. Fourteen lines fell within the Williams 82-like haplogroup, but only one of these lines, C08, was a cultivated line (Figure 2). This broad haplogroup could be subdivided into two sub-groups (Figure 2). The 17 lines of the non-Williams 82 haplogroup included 13 cultivated lines but only four wild lines W05, W11, W13, and W14 (Figure 2). When assigning genotypes by looking at the causative SNP, the 12 *e2* lines, comprising ten cultivars and two wild, were part of the non-Williams 82 haplogroup (Figure 2, Tables 3 and 4). However, two wild lines (W13 and W14), two Chinese cultivated lines (C14 and C17), and one Taiwan mutagenized Japanese variety (C16) were also within this non-Williams 82 haplogroup, but these lines were *E2* (Figure 2, Tables 3 and 4).

**Figure 3:**
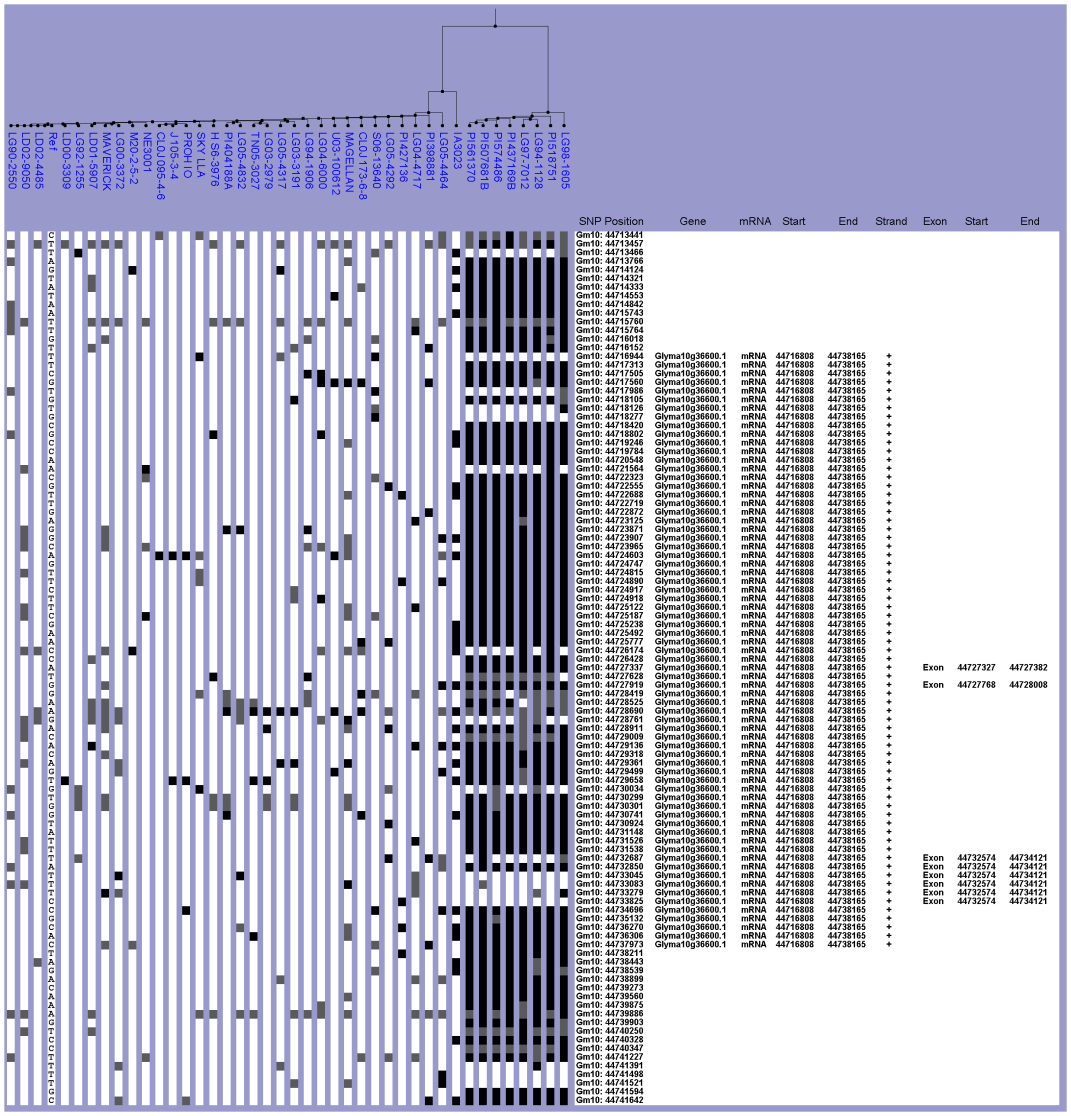
Haplotype analysis of the *E2* gene region for the NAM parents. The SNPViz clustering pictorial displayed the SNPs in a 28.3-kb region on chromosome ten, which included Glyma10g36600. Nucleotide polymorphisms were examined in the NAM parents (blue). Details about the SNPViz clustering pictorial were described in the Figure 1 legend.

Thirty-three NAM parent lines were entirely Williams 82-like except for some very limited base changes from the reference and some positions with no nucleotide information (Figure 3). The other eight NAM parents PI561370, PI507681B, PI574486, PI437169B, LG97-7012, LG94-1128, PI518751, and LG98-1605 all belonged to the non-Williams 82 haplogroup (Figure 3). Examination of the causative SNP indicated that the 33 lines grouped with Williams 82 also shared the *E2* allele while the non-Williams 82 lines were all *e2* (Table 5).

The allelic distribution of *E2* and *e2* as well as the haplotypes varied between the Chinese cultivars and the NAM and wild lines. Comparison of the Chinese collection and NAM lines side by side in the same haplotype region grouped all *e2* lines in the non-Williams 82 haplogroup (Figure S3). This haplogroup was subdivided, and one subgroup contained all eight of the *e2* NAM lines while the other subgroup contained 13 cultivated and four wild lines of which three cultivated lines were *E2* [two lines popularized outside of China and a southern landrace (C17)]. Only two wild lines W11 and W05, both from southern China (Shanxi and Henan), had the *e2* allele, but two other wild lines originating in Henan were *E2*.

### *E3* Haplotype Characterization

In our two datasets, we analyzed the allelic variation of the photochrome A gene *E3* because *E3* contributes to delayed flowering under long-day conditions, which ultimately affects maturity [14]. Glyma19g41210 is an 8958 bp gene with four exons and encodes an 1170 amino acid protein (Glyma1.1, http://www.phytozome.net/). Two functional alleles, *E3* and *E3 (short)*, differ only by a 2.6-kb deletion in the third intron (Table 2) [7]. The nonfunctional *e3* allele is a result of a 15.5-kb deletion (compared to the Williams 82 *E3* allele) leading to a premature stop codon [7] (Table 2). The other mutant *e3-ft3* allele, which causes an amino acid change of G1050R, was not found in our datasets [7]. In order to not rely completely on identification of deletions in SNP-array datasets, the Chinese collection and NAM lines were characterized by haplogroups and also by utilizing a new genotyping assay for the NAM parent lines that distinguished *E3*, *E3 (short)*, and *e3* alleles. The haplotype viewing region (44,713,304–44,741,665 bp) included the entirety of the *E3* gene region and 3.5 kb on either side of the gene (Table 1).

**Figure 4:**
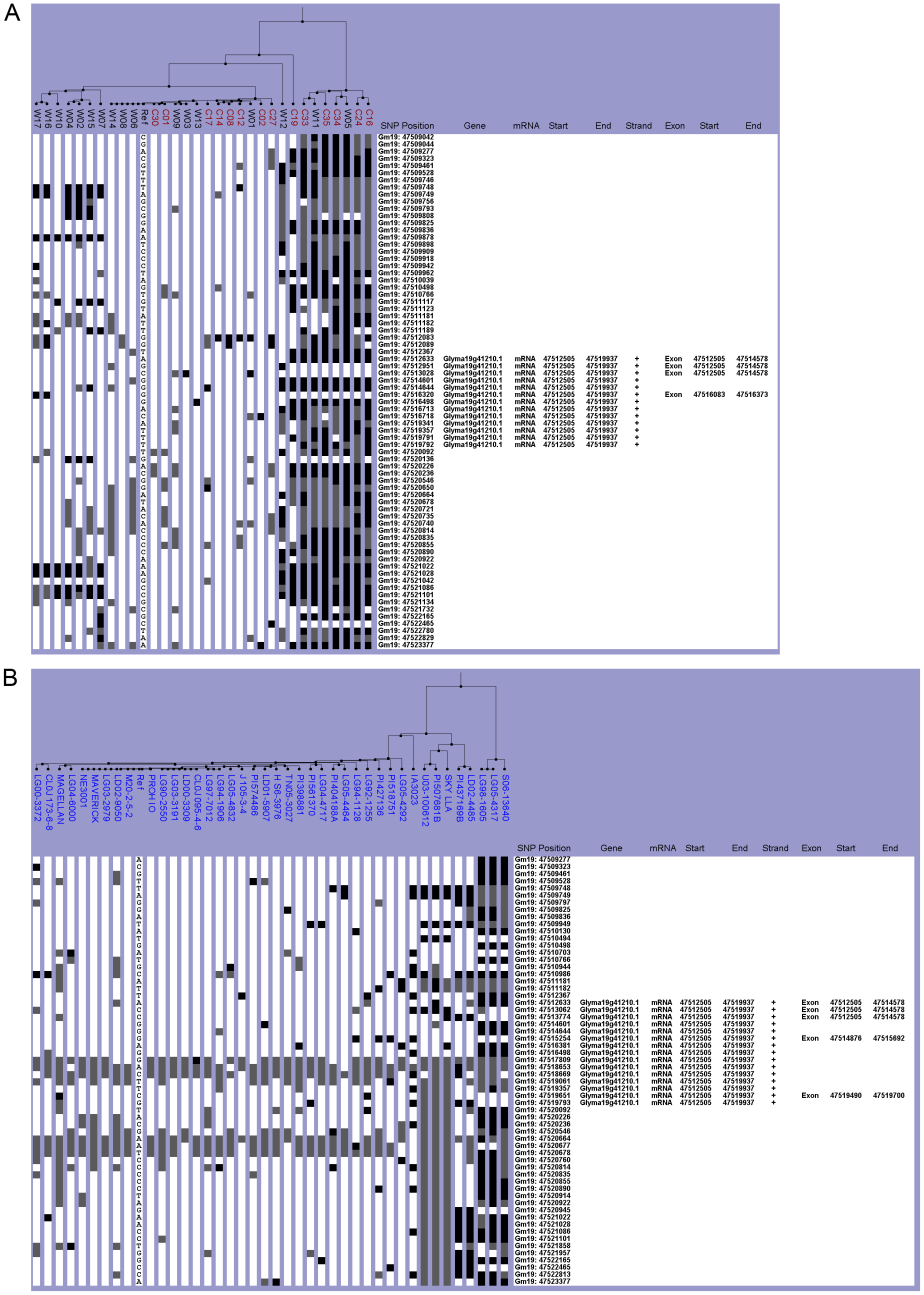
Haplotype analysis of the *E3* gene region. The SNPViz clustering pictorial displayed the SNPs in a 14.4-kb region on chromosome 19, which included Glyma19g41210. Nucleotide polymorphisms were examined in A) the wild (black) and cultivated (red) lines from the Chinese collection and B) the NAM parents (blue). Details about the SNPViz clustering pictorial were described in the Figure 1 legend.

In the Chinese collection, 23 lines, including nine cultivars and 15 wild lines, were clustered in the same haplogroup as Williams 82 (Figure 4A). Five cultivated lines and two wild lines were grouped in the non-Williams 82 haplogroup (Figure 4A). Even though lines were categorized into haplogroups based on similarity or dissimilarity to the reference, lines belonging to the Williams 82-like haplogroup could not be described as having the *E3* allele simply because Williams 82 was *E3*. Genotypes could not be assigned since a causative SNP was not present. Also, genotyping the Chinese collection directly was not an option because DNA was unavailable.

The majority of the NAM parents fell within the Williams 82-like haplogroup while only three lines were clustered in the non-Williams 82 haplogroup (Figure 4B). In the Williams 82-like haplogroup, Skylla, PI507681B, and U03-100612 had a similar yet distinctive haplotype compared to the reference (Figure 4B). These lines had no SNP data from base position 47,517,809 within intron three to the end of the haplotype region (Figure 4B). This lack of data was consistent with the position of the *e3* 15-kb deletion in intron three. To confirm whether Skylla, PI507681B, and U03-100612 had the *e3* allele, a PCR genotyping assay was developed to distinguish *E3*, *E3 (short)*, and *e3* alleles. Genotyping the *E3* alleles was possible because DNA was available for the NAM parents (Table 5). This PCR analysis confirmed that Skylla, PI507681B, and U03-100612 were *e3*. This assay also revealed that LG98-1605, S06-13640, and LG05-4317 were *E3 (short)*. The *E3 (short)* lines were the only lines that belonged to the non-Williams 82 haplogroup. The NAM parent lines with *E3* alleles all had two regions of fairly consistent lack of data (Figure 4B). One of these regions (47,517,809–47,518,669 bp) roughly corresponded to the position of the 2.6 kb-difference in alleles between the Williams 82 *E3* and *E3 (short)*. The three confirmed *E3 (short)* lines also had missing data for most of this region (Figure 4B).

Despite the fact that the *E3* alleles could not be exactly determined in the Chinese collection, the haplogroups were compared directly to the NAM dataset. When these two datasets were integrated into the same haplotype analysis, the haplogroups were comparable to the individual dataset analysis (Figure S4). The seven Chinese lines originally in the non-Williams 82 haplogroup categorized with the three *E3 (short)* lines from the NAM parents. Based on the absence of the missing data region, we could not confidently assign any of the Chinese lines the *e3* genotype.

### *E4* Haplotype Characterization

Another phytochrome gene, *E4*, was studied for allelic variation because it also involved flowering time delay [14]. Glyma20g22160 is a 5,895 bp gene with four exons and encodes an 1123 amino acid protein (Glyma1.1, http://www.phytozome.net/). In addition to the functional *E4* allele, five dysfunctional alleles, *e4* (*SORE-1*), *e4-oto*, *e4-tsu*, *e4-kam*, and *e4-kes*, have been identified [15]. Examining a causative SNP for these alleles was not possible because *e4* (*SORE-1*) has an insertion of a *Ty1/copia*-like retrotransposon in exon one, and each of the other four alleles has a single-base deletion in a gene-coding region [8],[15] (Table 2). Since we could not determine which *e4* nonfunctional alleles were present in the Chinese collection and NAM parents, we characterized the haplogroups by examining the *E4* gene region as well as 3.5 kb surrounding the gene (32,084,161–32,096,766 bp) (Table 1).

**Figure 5:**
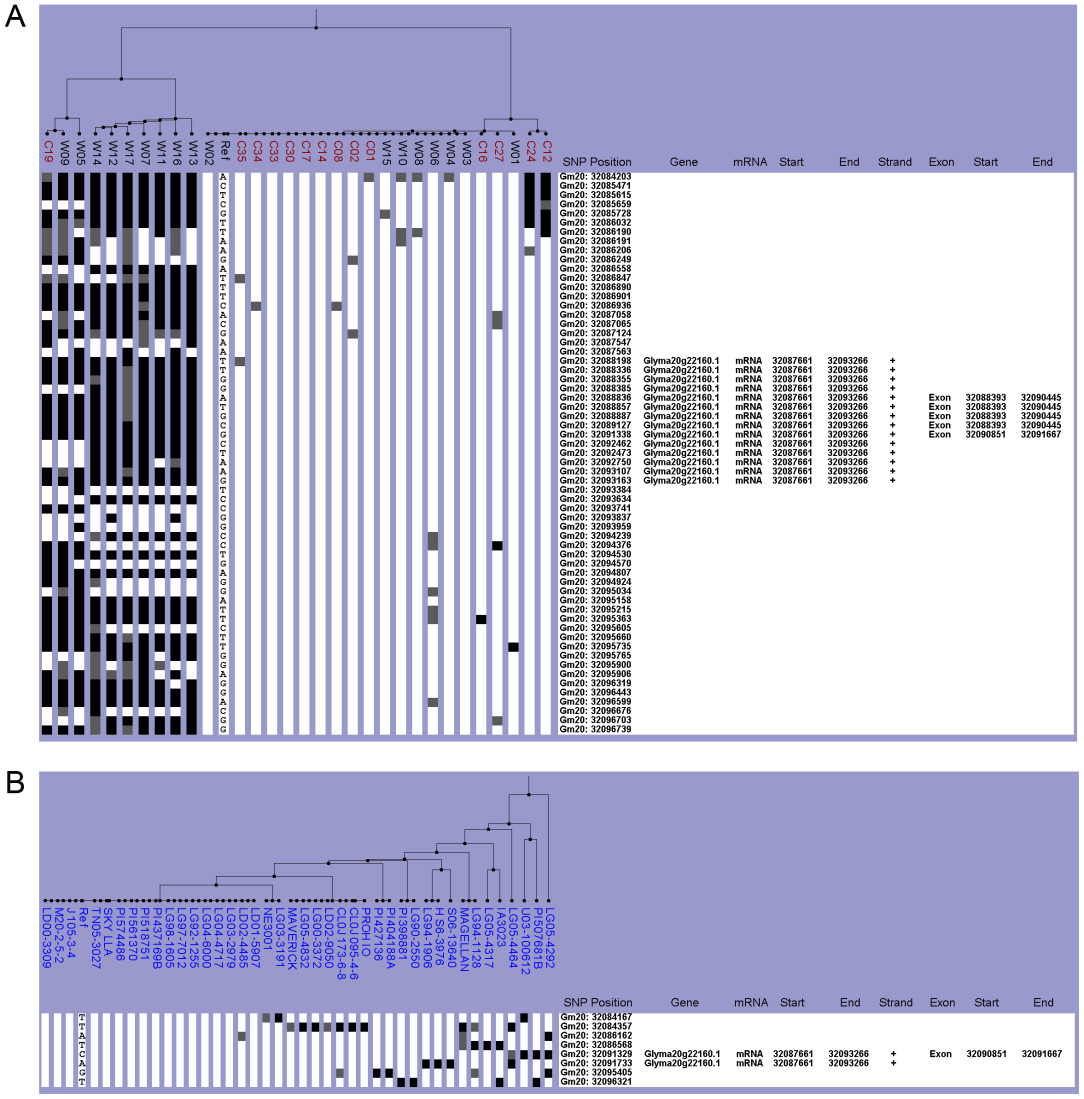
Haplotype analysis of the *E4* gene region. The SNPViz clustering pictorial displayed the SNPs in a 12.6-kb region on chromosome 20, which included Glyma20g22160. Nucleotide polymorphisms were examined in A) the wild (black) and cultivated (red) lines from the Chinese collection and B) the NAM parents (blue). Details about the SNPViz clustering pictorial were described in the Figure 1 legend.

Within this region, lines were either completely like Williams 82 or distinctively different from Williams 82. Twenty-one of the Chinese lines were grouped with Williams 82, which has the *E4* functional allele (Figure 5A). Seven of the lines were wild while the other 14 were cultivated lines (Figure 5A). The remaining ten lines could be classified into a separate haplogroup that was non-Williams 82-like. All lines within this haplotype group were wild except for C19 (Figure 5A). However, the NAM parents all belonged to a single Williams 82-like haplogroup, which suggested that the NAM parents have the *E4* allele (Figure 5B). When both datasets were integrated into one analysis, all lines were categorized into the same haplogroups as they did in the individual dataset analysis (Figure S5). Not surprisingly, only ten of the 72 lines were grouped in the non-Williams 82 haplotype, which was consistent with the fact that the various *e4* alleles have essentially been found in relatively small geographical locations in high latitudes of Eastern Asia [15],[23].

### *Dt1* Haplotype Characterization

We surveyed the allelic variation for *Dt1* in the Chinese and NAM datasets because plant architecture is an important trait in soybean related to photoperiod flowering response. Glyma19g37890 is a 1915 bp gene with four exons and encodes a 173 amino acid protein, alternatively named *GmTf1-a* (Glyma1.1, http://www.phytozome.net/) [9]. The gene is oriented on the chromosome in the antisense direction relative to increasing chromosome nucleotide position. In addition to the two functional *Dt1* alleles with identical amino acid sequences, four missense *dt1* alleles*—dt1* (R62S), *dt1* (P113L), *dt1* (R130K), and *dt1* (R166W)—have been reported to condition a determinate plant architecture phenotype [9],[10]. These four *dt1* alleles each have a causative SNP position where one nucleotide is substituted for another in the gene-coding region (Table 2). Only *dt1* (R62S), *dt1* (P113L), and *dt1* (R166W) appeared in our datasets. These *dt1* alleles were identified by the causative SNP location 44,981,190 bp (C/A), 44,980,245 (G/A), and 44,980,087 (T/A), respectively (Table 2).

**Figure 6:**
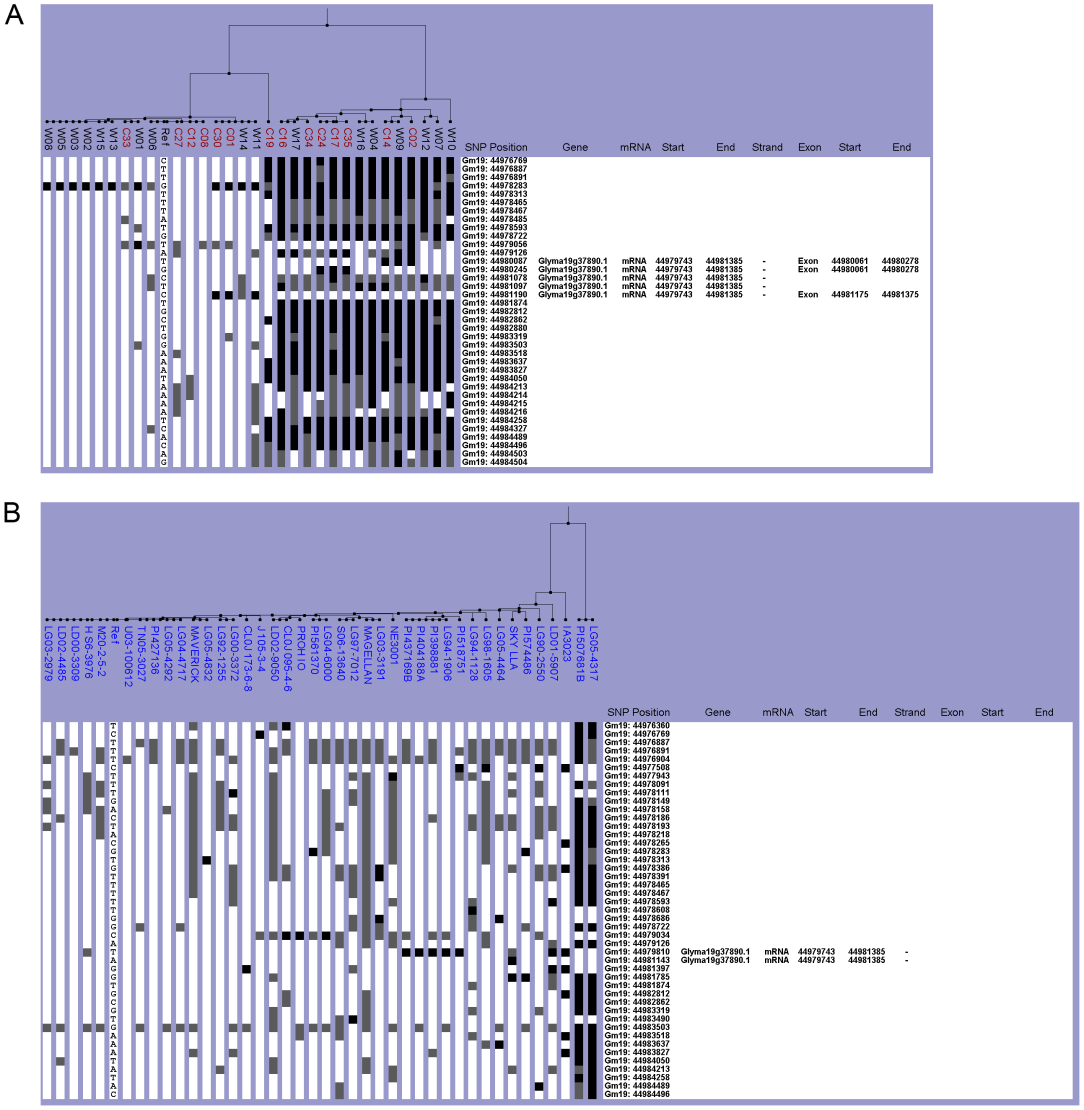
Haplotype analysis of the *Dt1* gene region. The SNPViz clustering pictorial displayed the SNPs in an 8.6-kb region on chromosome 19, which included Glyma19g37890. Nucleotide polymorphisms were examined in A) the wild (black) and cultivated (red) lines from the Chinese collection and B) the NAM parents (blue). Details about the SNPViz clustering pictorial were described in the Figure 1 legend.

The Williams 82-like and non-Williams 82 haplogroups were almost evenly divided in the Chinese collection. The Williams 82-like haplogroup contained 17 lines while the non-Williams 82 haplogroup had 14 (Figure 6A). Of the 17 William 82-like lines, ten were wild and seven were cultivated (Figure 6A). The non-Williams 82 lines were comprised of an equal number of wild and cultivated lines. By looking at the causative SNPs, nine lines were identified as having one of the *dt1* alleles: *dt1* (R62S), *dt1* (P113L), or *dt1* (R166W). C02, C14, and W09 are *dt1* (R166W), and C17, C24, and C35 are *dt1* (P113L) (Tables 3 and 4). Three lines C01, C30, and W11 had the causative SNP for *dt1* (R62S) (C/A at 44,981,190 bp), but a fourth line W14 was in the same subgroup (Figure 6A, Tables 3 and 4). Since W14 had no data at this base position, it could not be classified as *dt1* (R62S). The *dt1* (P113L) and *dt1* (R166W) lines fell within the non-Williams 82 haplogroup, but the *dt1* (R62S) lines were grouped with Williams 82 (Figure 6A).

All of the NAM parent lines were classified as indeterminate *Dt1*. The majority (38 lines) of the NAM parents were clustered in the Williams 82-like haplogroup. The remaining two lines PI507681B and LG05-4317 were within the non-Williams 82 haplogroup (Figure 6B). None of the *dt1* causative SNPs were found in the NAM dataset; therefore, all NAM lines were *Dt1* (Table 5). When the datasets were analyzed together, the two major haplogroups were consistent with the individual datasets (Figure S6). The two NAM parent lines that were not in the Williams 82-like haplogroup (PI507681B and LG05-43170) formed a distinct subgroup when the two datasets were combined.

## Discussion

The development of software to create phylogenetic trees of soybean lines and pictorial representations of haplotypes based on SNP-array data from whole genome resequencing enabled us to examine the haplotype diversity and allelic variation for soybean genes related to the photoperiod response. Using just the first tier of haplotype grouping for our five genes of interest, we were able to determine that two haplogroups either had a high degree of nucleotide identity with the Williams 82 reference sequence or had a high degree of sequence dissimilarity with the reference sequence. For each gene, the set of soybean lines that formed the two groups was unique, and overall, little evidence was seen for artificial selection for these genes that separated the wild *Glycine soja* lines from the cultivated *Glycine max* lines. Within each major group, the lines were also separated into subgroups that further revealed haplotype diversity.

We used two datasets that presented us with soybean accessions that had contrasting population structures. The Chinese lines consisted of wild soybeans, Chinese landraces, Chinese improved lines, and single lines from Japan (fast neutron mutagenized and used in Taiwan), Brazil, and the USA. The NAM parent lines were selected based on nomination from cultivars, experimental lines, and plant introductions with similar maturity (maturity groups II, III, and IV) by the public soybean breeding community in the USA. The final selection for NAM parent lines was based on maximizing genetic diversity from that group and included 17 high yielding parents, 15 lines with diverse ancestry, and eight plant introduction lines (B. Diers, personal communication 2013). By examining each dataset independently and as an integrated set with SNPViz, we were able to develop a better understanding of allele diversity for the *E1*, *E2*, *E3*, *E4*, and *Dt1* genes. For each gene, multiple wild soybean lines clustered in both haplogroups. This result suggests purifying selection during soybean domestication did not occur or that continued gene flux has been a factor during landrace selection and improved cultivar development for these genes. The exception was for *E4*, which showed very little nucleotide diversity for all cultivated lines (only one cultivated line from the Chinese collection was in the non-Williams 82 haplogroup, which contained nine wild lines).

Additional sequence data is being generated that can be incorporated into our analysis, but more research is required to determine an optimum dataset that allows the discovery of the major haplotype groups for populations with defined structures (wild, landraces, elite varieties, and wide geographic distribution) [24]. Since all the SNPs are unweighted, the SNPViz analysis is not necessarily able to separate haplotypes by causative mutations unless the defining SNP position is used as the search query. However, the SNPViz analysis did provide information on genetic context in which mutant alleles likely arose.

For both datasets for *E1*, *E2*, and *Dt1*, we were able to unambiguously assign a functional or mutant allele of each gene for each soybean line in the analysis because the causative SNP was present in the data (Tables 3 and 4). Determining the correlation of those alleles with line type and also with haplogroups was possible. In the Chinese collection, where the soybeans were selected across major geographic regions differing in typical growing season length as well as latitude, the predominant allele in both wild and cultivated lines was *E1. E2* was most prevalent in the wild lines while *e2* predominated in the cultivated lines. *Dt1* was present almost exclusively in the wild lines, but the cultivated lines were a mixture of *Dt1* and three *dt1* alleles. In contrast, for the NAM parent lines, *e1-as*, *E2*, and *Dt1* predominated. This result indicated contrasting selection for *E1* and *e2* for the Chinese cultivated soybean lines and *e1-as* and *E2* for the majority of the NAM parents. The only cultivated *e1-as* line C08 from the Chinese collection was actually the line ‘Union’ [25] related to Williams 82 and developed in the USA. The three wild lines containing *e1-as* were all collected from the northeast part of China in the Heilongjian province, and those three wild lines clustered with the Williams 82 haplogroup. We hypothesized that the *e1-as* allele arose in the wild soybean population in Heilongjian and was subject to artificial selection for early maturity that proved to be essential for development of productive early maturing lines adapted to North American soybean growing areas.

The SNPViz software tool has the ability to produce a visual representation and phylogenetic tree of a chromosome region or allele haplotype from whole genome sequencing datasets. While we are particularly interested in soybean, the software can be applied to datasets from any organism, although obviously lower quality sequence data may prevent meaningful analysis. In each of our target genes, two broad haplogroups were revealed compared to the reference sequence, but analysis of gene regions with multiple haplogroups may prove interesting. We were able to identify possible gene flow and potential geographic origin of alleles of genes, particularly for *E1* and *e1-as*. Previous geographic analysis of the allele diversity of the *Dt1* gene [9] was consistent with our findings, and we were able to identify two of the four *dt1* mutant alleles present in wild lines (the R62S and R166W mutations).

SNPViz is an innovative software program that can be used to study allelic variation and diversity for specific chromosome regions and known and predicted genes. We have demonstrated that SNPViz is capable of handling a haplogroup analysis of genes with causative SNPs, such as *E1*, *E2*, and *Dt1*, as well as different mutations, as shown with *E3* and *E4*. The web-based and stand-alone version of the SNPViz software is available at SoyKB (http://soykb.org/SNPViz). We strongly encourage other researchers to apply this SNPViz software to their own whole genome sequencing SNP analyses.

## Supporting Information

Figure S1 **Example of SNPViz output displays for the** ***Dt1*** **gene region.** SNPViz offers the user four visual output options. For all options, the reference nucleotides are always displayed. As shown here, users can include an annotation file so that the gene location, exon positions, and DNA strand information are included in the pictorial. However, users can decide to not include an annotation file, and gene information will not be presented next to the base positions. A) Nucleotides can be represented by three colors. Black indicates that a base at that position is different from the reference sequence. White means that the base at that position is identical to the reference. The nucleotide is gray if data is not present or missing. B) The three-color option can also include SNP nucleotides shown for every sample. C) SNP data can also be represented by different colors, where red, blue, green, and yellow are used to indicate A, G, C, and T, respectively. D) The multiple color option can also include the nucleotides shown for every sample. (TIF)

Figure S2 **The integrated haplotype analysis of the** ***E1*** **gene region.** The SNPViz clustering pictorial displayed the SNPs in a 7.8-kb region on chromosome six, which included Glyma06g23026. The nucleotide polymorphisms were examined by integrating the Chinese wild (black), cultivated (red), and NAM parents (blue) datasets together for one analysis. (TIF)

Figure S3 **The integrated haplotype analysis of the** ***E2*** **gene region.** The SNPViz clustering pictorial displayed the SNPs in a 28.3-kb region on chromosome ten, which included Glyma10g36600. The nucleotide polymorphisms were examined by integrating the Chinese wild (black), cultivated (red), and NAM parents (blue) datasets together for one analysis. (TIF)

Figure S4 **The integrated haplotype analysis of the** ***E3*** **gene region.** The SNPViz clustering pictorial displayed the SNPs in a 14.4-kb region on chromosome 19, which included Glyma19g41210. The nucleotide polymorphisms were examined by integrating the Chinese wild (black), cultivated (red), and NAM parents (blue) datasets together for one analysis. (TIF)

Figure S5 **The integrated haplotype analysis of the** ***E4*** **gene region.** The SNPViz clustering pictorial displayed the SNPs in a 12.6-kb region on chromosome 20, which included Glyma20g22160. The nucleotide polymorphisms were examined by integrating the Chinese wild (black), cultivated (red), and NAM parents (blue) datasets together for one analysis. (TIF)

Figure S6 **The integrated haplotype analysis of the** ***Dt1*** **gene region.** The SNPViz clustering pictorial displayed the SNPs in an 8.6-kb region on chromosome 19, which included Glyma19g37890. The nucleotide polymorphisms were examined by integrating the Chinese wild (black), cultivated (red), and NAM parents (blue) datasets together for one analysis. (TIF)
