# Supplementary figures and images for: Major Soybean Maturity Gene Haplotypes Revealed by SNPViz Analysis of 72 Sequenced Soybean Genomes
Source: PLoS One. 2014 Apr 11;9(4):e94150. doi: 10.1371/journal.pone.0094150 (PMC3984090; doi:10.1371/journal.pone.0094150)

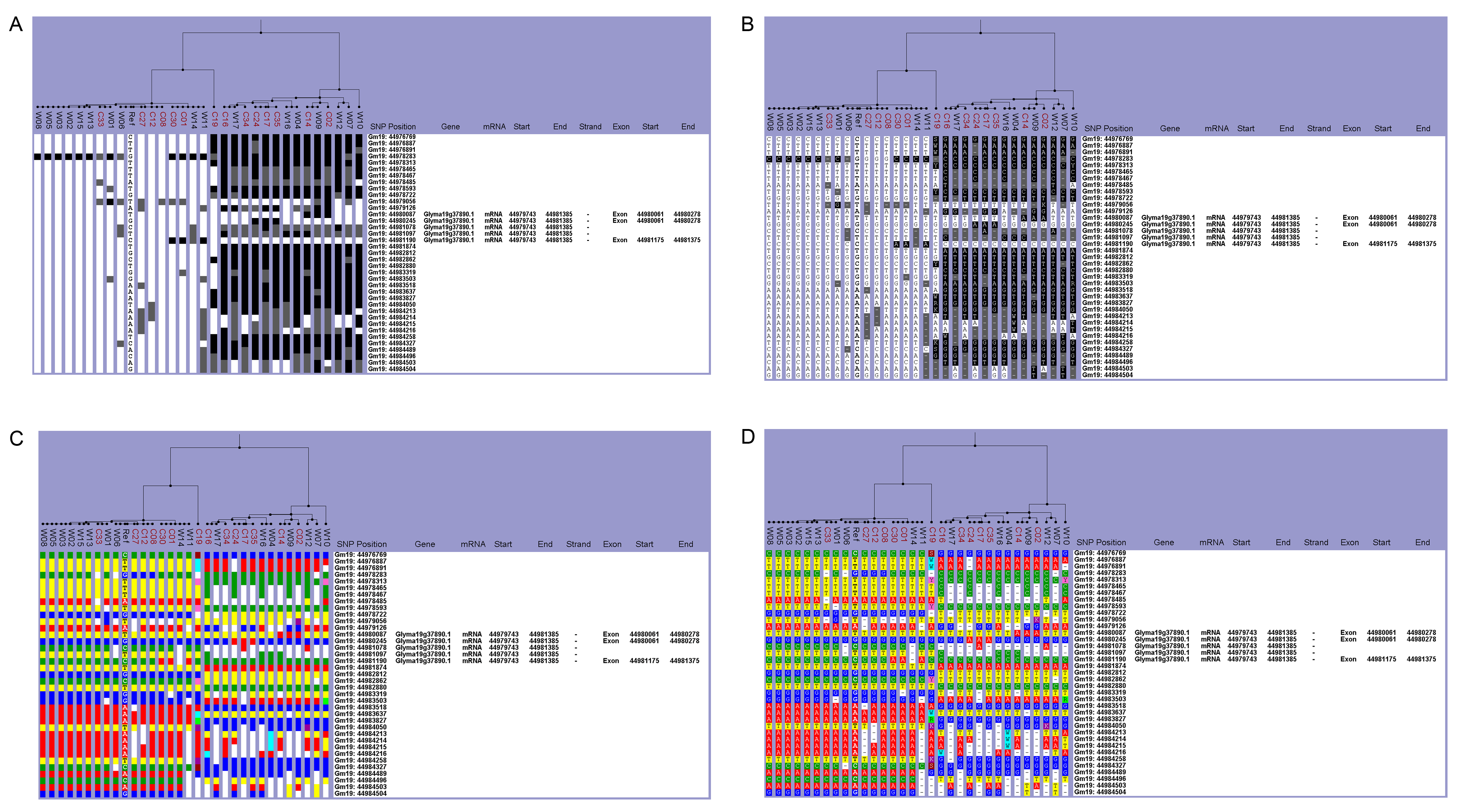

Supplement: Figure S1 — (TIF) [file pone.0094150.s001.tif]

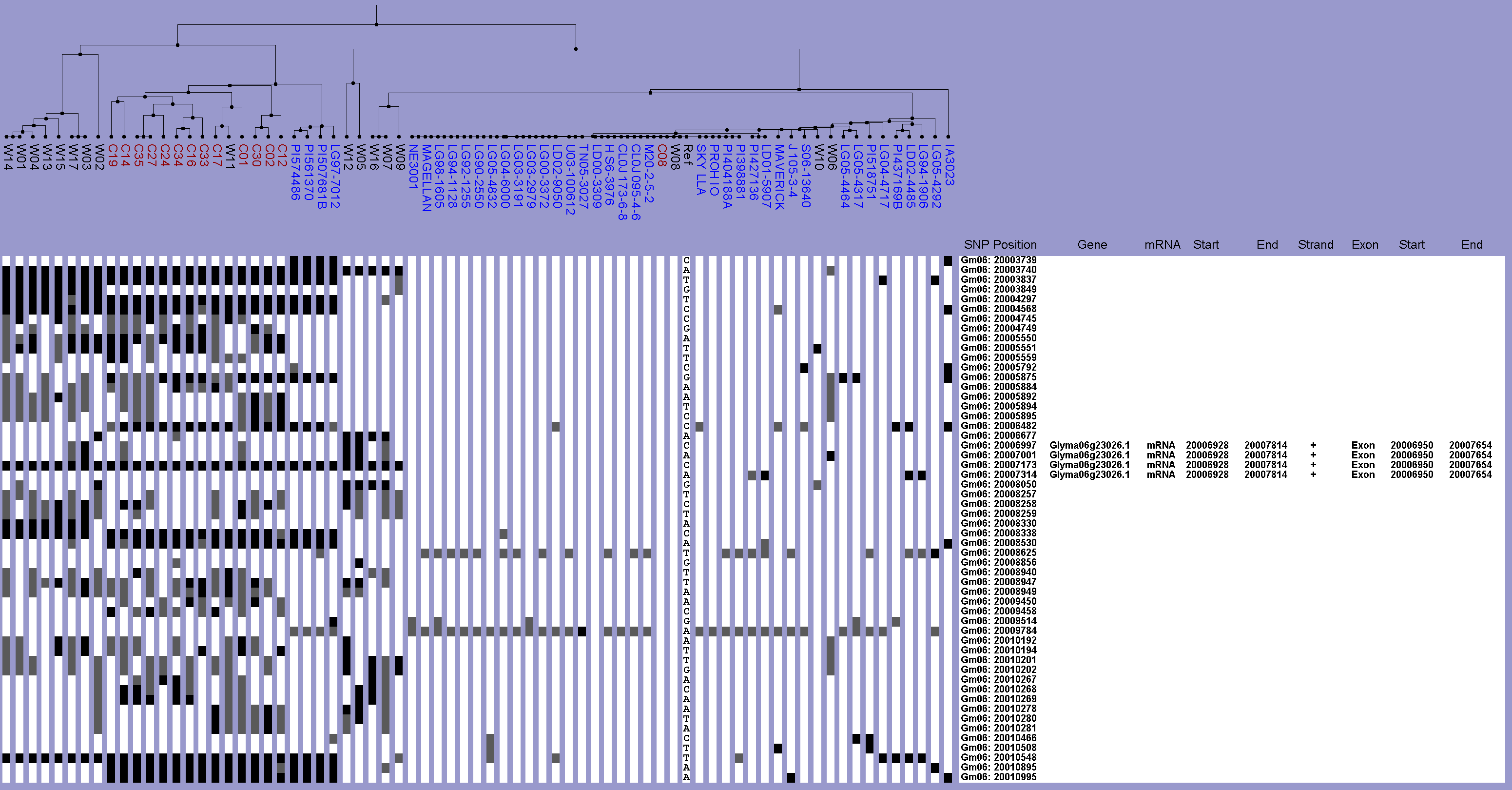

Supplement: Figure S2 — (TIF) [file pone.0094150.s002.tif]

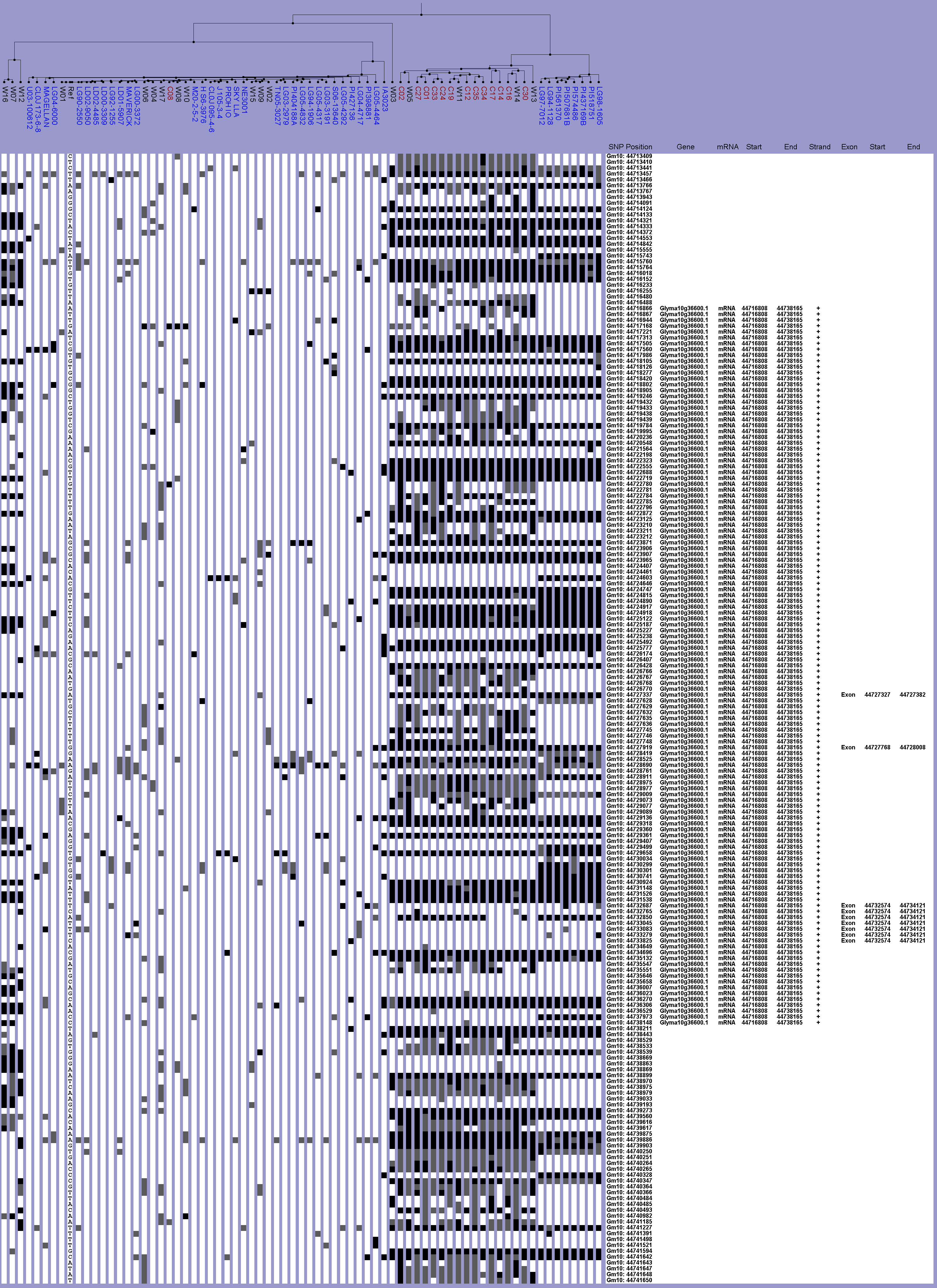

Supplement: Figure S3 — (TIF) [file pone.0094150.s003.tif]

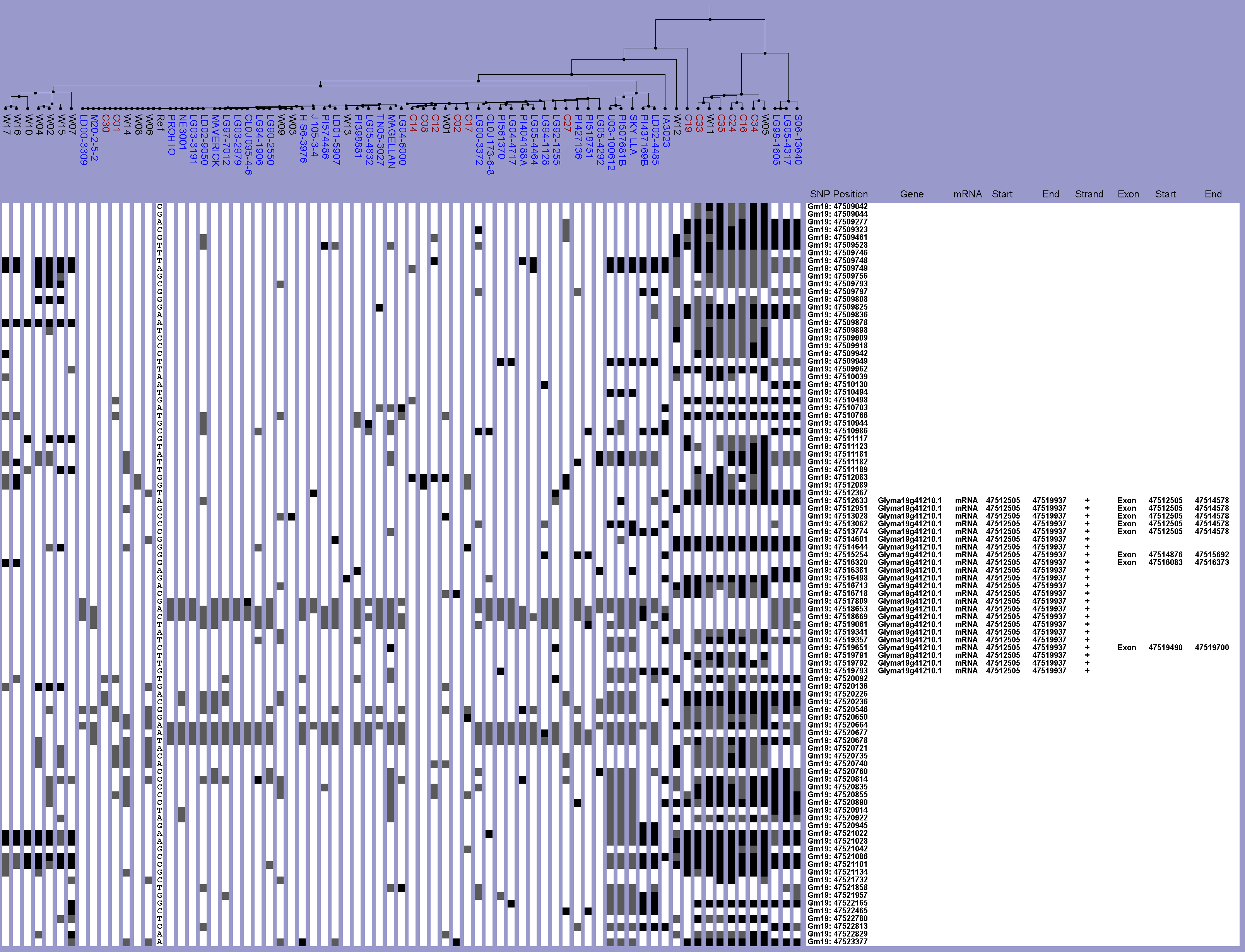

Supplement: Figure S4 — (TIF) [file pone.0094150.s004.tif]

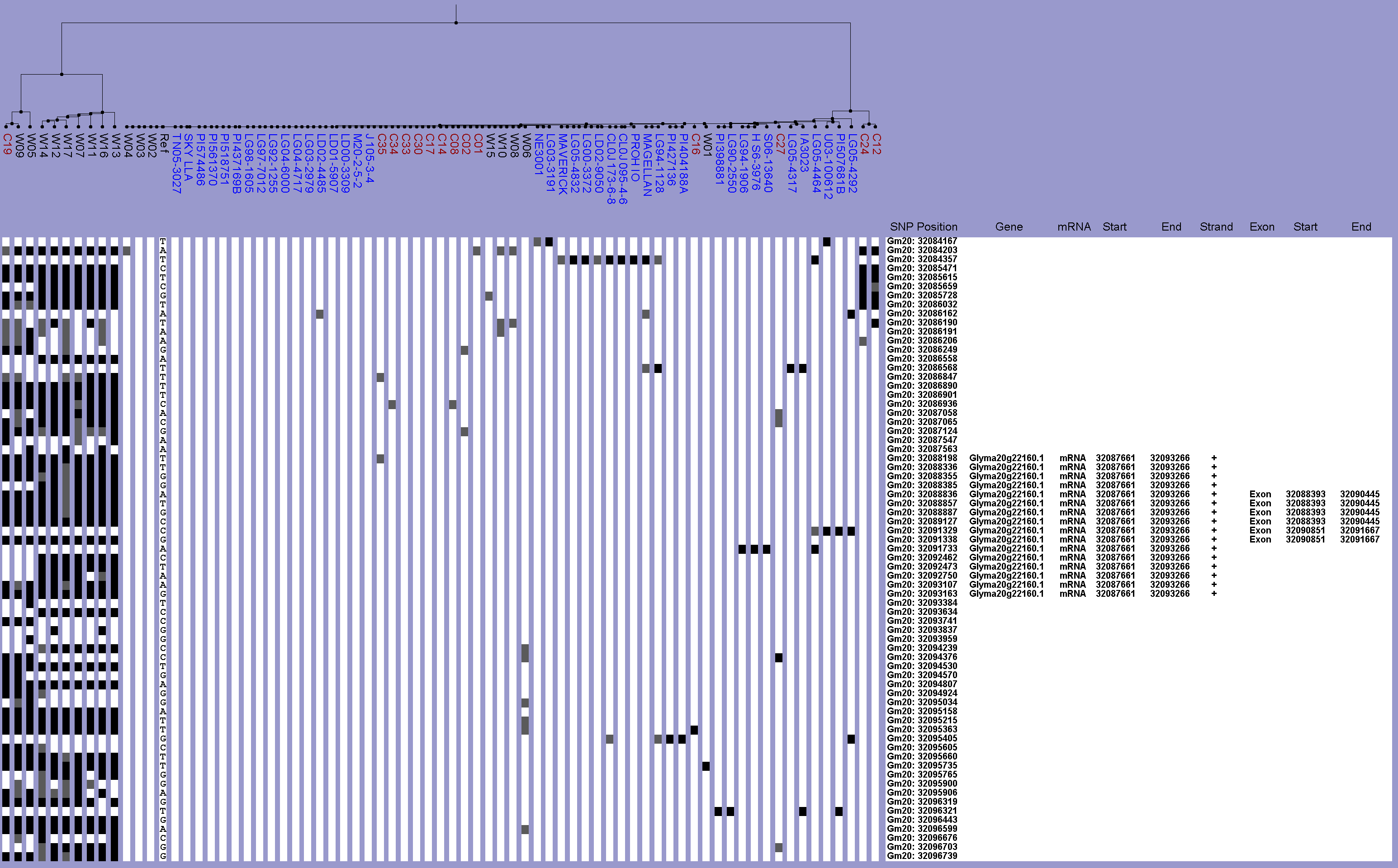

Supplement: Figure S5 — (TIF) [file pone.0094150.s005.tif]

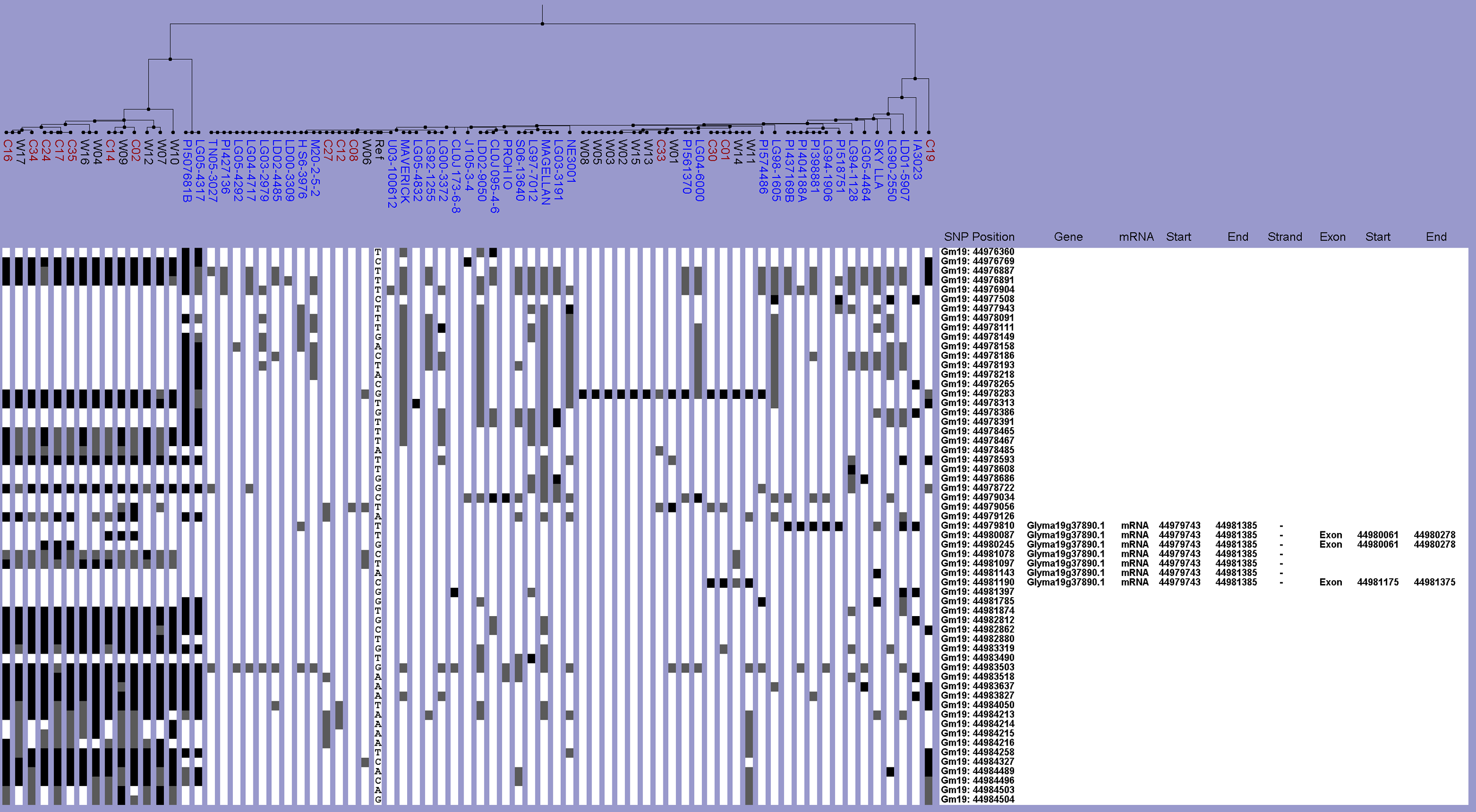

Supplement: Figure S6 — (TIF) [file pone.0094150.s006.tif]
